# Activin-A signaling promotes epithelial–mesenchymal transition, invasion, and metastatic growth of breast cancer

**DOI:** 10.1038/npjbcancer.2015.7

**Published:** 2015-08-12

**Authors:** Mohsin Bashir, Surekha Damineni, Geetashree Mukherjee, Paturu Kondaiah

**Affiliations:** 1 Department of Molecular Reproduction, Development and Genetics, Indian Institute of Science, Bangalore, India; 2 Department of Pathology, Kidwai Memorial Institute of Oncology, Bangalore, India

## Abstract

**Background:**

Activins belong to the transforming growth factor-β (TGF-β) superfamily of cytokines. Although the role of TGF-β in cancer progression has been highly advocated, the role of activin signaling in cancer is not well known. However, overexpression of activin-A has been observed in several cancers.

**Aims:**

The gene expression profile indicated higher expression of Activin-A in breast tumors. Hence the aim of this study was to evaluate the status and role of Activin signaling pathway in these tumors.

**Methods:**

Microarray analysis was performed to reveal gene expression changes in breast tumors. The results were validated by quantitative PCR and immunohistochemical analysis in two independent sets of normal and tumor samples. Further, correlation of activin expression with survival and distant metastasis was performed to evaluate its possible role in tumor progression. We used recombinant activin-A, inhibitors, overexpression, and knockdown strategies both *in vitro* and *in vivo*, to understand the mechanism underlying the protumorigenic role of this signaling pathway.

**Results:**

We report that activin-A signaling is hyperactivated in breast cancers as indicated by higher activin-A, phosphoSMAD2, and phosphoSMAD3 levels in advanced breast cancers. Bone morphogenetic proteins and molecules involved in this signaling pathway were downregulated, suggesting its suppression in breast cancers. Activin-A expression correlates inversely with survival and metastasis in advanced breast cancers. Further, activin-A promotes anchorage-independent growth, epithelial–mesenchymal transition, invasion, angiogenesis, and stemness of breast cancer cells. We show that activin-A-induced phenotype is mediated by SMAD signaling pathway. In addition, activin-A expression affects the tumor-forming ability and metastatic colonization of cancer cells in nude mice.

**Conclusions:**

These results suggest that activin-A has a critical role in breast cancer progression and, hence, targeting this pathway can be a valuable strategy in treating breast cancer patients.

## Introduction

Activins are members of the transforming growth factor-β (TGF-β) superfamily of cytokines, which includes TGF-β, activins, nodal, inhibins, growth and differentiation factors (GDFs), and bone morphogenetic proteins (BMPs).^1,2^ Activin-A binds to type II transmembrane serine–threonine kinase receptor (ActRIIA or ActRIIB), which in turn activates type I receptor (ActRIB), leading to phosphorylation of SMAD2/SMAD3. On phosphorylation, SMAD2/3 forms a complex with SMAD4, which then translocates to the nucleus. In the nucleus, Smad2/3/4 complex along with other co-factors regulate the expression of a large number of genes.^3^ Activins were initially shown to have an important role in gonadal function.^4^ Subsequently, they have been shown to have an important role in gonadal function,^5^ embryonic development,^6^ pancreatic function,^7^ bone formation,^8^ mammary gland development,^9^ cell proliferation,^10^ maintenance of embryonic stem cells,^11^ and immune response.^12^

Activin-A has been shown to be overexpressed in metastatic prostate cancer,^13^ stage 4 colorectal cancers,^14^ lung cancer,^15^ hepatocellular carcinomas,^16^ and pancreatic cancers.^17^ Activin signaling has also been shown to promote aggressiveness of esophageal squamous cell carcinoma cells^18^ and skin tumorigenesis.^19^ Interestingly, inhibin-α (activin antagonist)-deficient mice have been shown to develop gonadal and adrenal cortical tumors.^20^ The involvement of activin signaling in cancer cachexia has also been advocated.^21^ Activin-A has been shown to induce a protumorigenic phenotype by facilitating tumor cell–tumor microenvironment interactions, leading to increased levels of cytokine production and cell motility.^22^

The role of activins in breast cancer progression is not well studied. Earlier, downregulation of activin signaling in breast tumors has been reported.^23^ On the contrary, increased serum level of activin has been reported in women with breast cancers.^24^ We performed global differential expression of genes in breast cancers with respect to normal tissue samples. The data revealed *INHBA* to be one of the highly upregulated genes with no appreciable change in the expression of any TGF-β isoforms. It is important to note that TGF-β, which has been implicated in the progression and metastatic spread of breast cancers, also functions through the same set of downstream effectors, Smad2 and 3. Hence, it becomes important to evaluate the role of activin-A in breast cancer progression. In this study we show that activin-A signaling pathway is activated in breast cancers and provide data that suggests its active role in breast cancer progression.

## Materials and methods

### Reagents

Recombinant human activin-A (338-AC-010) and activin-A antibody (AF338) were purchased from R&D Systems (Minneapolis, MN, USA); phosphoSMAD2 (3101 and 9510) from Cell Signaling Technology (Boston, MA, USA); SMAD3 (1735), E-cadherin (1702), N-cadherin (2019), and α-smooth muscle actin (1184-1) from Epitomics (CA, USA); Vimentin (V2258) and fluorescein isothiocyanate-conjugated phalloidin (P5282) from Sigma (St Louis, MO, USA); phosphoSMAD3 (ab52903) and BMP2 (ab14933) from Abcam (Cambridge, MA, USA); vascular endothelial growth factor-A (VEGF-A) (M7273) from Dako (Denmark); and PE-CD44 (560533)/PE-cy7 CD24 (555428) from BD (NJ, USA). The antibodies were used at a dilution of 1:100 or 1:200 for immunohistochemistry and most of the antibodies were used at a dilution of 1:1,000 for western blotting. Small hairpin RNA against activin-A is from Dharmacon (Lafayette, CO, USA) and small hairpin RNA for SMAD3 was a kind gift from Dr Lalage Wakefield. *INHBA* was overexpressed in mammalian expression pcDNA3.1 vector. The Student’s *t*-test was conducted to calculate the *P*-value for individual experiments and the median values plotted.

### Tissue samples and immunohistochemistry

Normal and tumor tissue samples were procured after obtaining informed consent of patients who underwent surgery at the Kidwai Memorial Institute of Oncology, Bangalore. The study has been approved by the Institute Ethical Committee of Kidwai Memorial Institute of Oncology. Details about tissue samples and expression analysis are listed with the Gene Expression Omnibus accession number GSE40206. All the patient details including histopathology data for expression of estrogen receptor, progesterone receptor, Her2/neu, tumor stage/grade, size, and lymph node status were documented for each case. The normal tissue samples were taken from non-tumor-bearing individuals, whereas all the tumor tissues belonged to grade III category. Staining was performed using the standard method of deparaffinization and gradual rehydration, followed by heat-mediated antigen retrieval in tris-EDTA or citrate buffer. The sections were further treated with 3% hydrogen peroxide in methanol to block endogenous peroxidase activity. Primary antibody incubation was done at room temperature for 2 h followed by incubation with an appropriate horseradish peroxidase-conjugated secondary antibody for 30 min. Detection was carried out using supersensitive polymer-horseradish peroxidase immunohistochemistry/diaminobenzidine detection system. The staining for different proteins was performed using the same set of tumor and normal samples. Analysis was performed by the pathologist determining the staining intensity of each section (with minimum of 10% cells positive) and grading from 0 (no staining) to 3+ (highest intensity). The list of primers used in the study is provided in Supplementary Table S1.

### Proliferation and soft agar assay

Proliferation of MCF-7 and MDA-MB-231 cells (from ATCC, Manassas, VA, USA) was assayed using bromodeoxyuridine cell proliferation assay kit (Calbiochem, San Diego, CA, USA) following the manufacturer’s protocol (24 h). For soft agar assay, 3,000 and 5,000 cells of MDA-MB-231 and MCF-7 (both from ATCC) were seeded for 12 and 18 days, respectively. The experiments were performed in triplicates for three independent times.

### Reporter assay

Matrix metalloproteinase-2 (MMP2) and VEGF reporter constructs were gifts from Etty Benveniste, University of Alabama, and Gail C. Fraizer, Kent State University, respectively. Luciferase reporter assays were carried out using promega kit (E4030, Madison, WI, USA) following the manufacturer’s protocol. Renilla luciferase under a TK promoter was used as an internal control. The experiment was performed two independent times in triplicates.

### Migration/invasion assay

For scratch assay, cells were cultured in a six-well plate till a confluent monolayer was formed. A scratch was made through the center of the well with a tip and images were recorded before and after activin-A treatment. Transwell migration and invasion assays were performed following the manufacturer’s protocol, using BD BioCoat Matrigel (NJ, USA) invasion/control chambers. The experiments were performed in triplicates and repeated two independent times. The images were recorded under a microscope with a fixed camera (×2 magnification) and cells were counted from several random fields.

### Zymography

Equal number of cells was seeded and the supernatant was collected after 24 h of serum starvation (stable clones) or activin-A treatment (parental cells). The sample was given a spin and equal volume was loaded into a non-denaturing gelatin containing gel (0.1%). The gel was incubated for 8–10 h followed by Coomassie staining. The images were recorded using a Uvipro platinum gel imaging system (Cambridge, UK).

### Mice experiments

For tumor xenograft growth, 5×10^5^ of MDA-MB-231 and 20×10^5^ MCF-7 cells were injected into the flank region of 4- to 5-week-old female nude mice. The animals were allocated by the animal facility and 10 animals were placed in each group randomly. Tumor formation was followed for 8 and 10 weeks, respectively. The tumors were excised and the weight of the tumors was plotted. To study metastatic spread of tumor cells, 20×10^5^ cells were injected into the tail vein of nude mice (5 animals each group). Mice were killed after 10 weeks of injection and various organs were analyzed for nodule formation. The size measurement was carried out on a Gatan Microscopy Suite (Gatan, Pleasanton, CA, USA), using a line scan that gives the number of pixels in a particular direction. The *t*-test was performed using Graphpad prism software (La Jolla, CA, USA) to evaluate the statistical significance of the data and a *P*-value above 0.05 was considered significant. All the experiments were performed in accordance with the institutional guidelines established for the Animal Facility at IISc and the study has approval from the Animal Ethics Committee of IISc.

## Results

### Activin-A signaling is active in breast cancer

**Figure 1:**
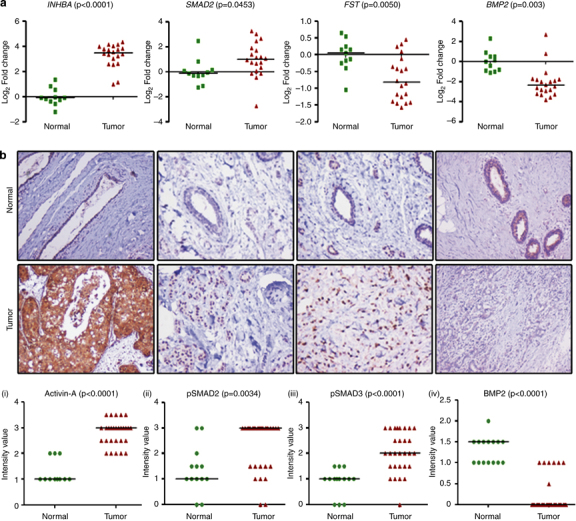

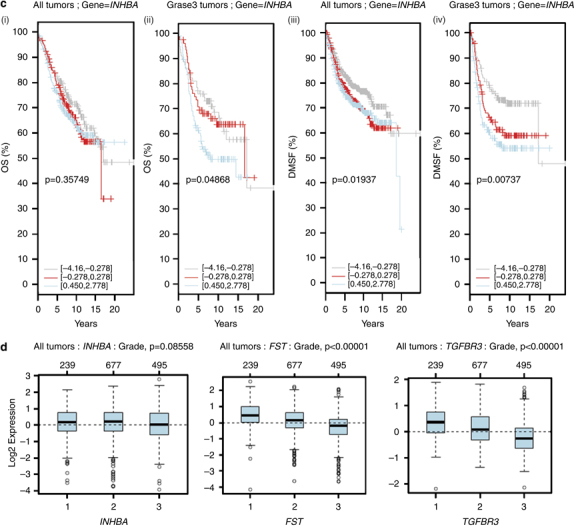
Expression of activin and correlation with breast tumor progression. (**a**) Quantitative PCR analyses of *INHBA*, *SMAD2*, *FST*, and *BMP2* expression in breast tumors compared with that in normal breast tissues. It is worth noting the significant increase in the expression of INHBA (activin-A) and Smad2. (**b**) Immunohistochemistry of activin-A (i), p*SMAD2* (ii), p*SMAD3* (iii), and *BMP2* (iv) in normal and breast tumor sections. Breast tumors show higher levels of activin-A, pSMAD2, and pSMAD3, whereas normal samples have higher levels of BMP2 compared with tumor samples. Each graph below shows the intensity score of individual normal and tumor sample on a scale of 0 to 3. The statistical significance is indicated in the representative graph. (**c**) GOBO gene set analysis shows that *INHBA* expression inversely correlates with overall survival (OS) of high-grade breast cancer patients (ii). In addition, *INHBA* expression correlates inversely with the distant metastasis-free survival (DMSF) of breast cancer patients (iii and iv). (**d**) GOBO box plot expression analysis shows that expression levels of negative regulators of activin signaling pathway, *FST* and *TGF*β*R3*, decrease progressively from grade 1 to grade 3.

Microarray study done in our laboratory revealed *INHBA* and various other genes involved in the activin signaling pathway to be differentially expressed in breast cancers (invasive ductal carcinoma) compared with normal tissue samples, suggesting activation of this signaling pathway (Supplementary Figure S1). This differential expression of the *INHBA* was not dependent on the category of breast tumors such as estrogen receptor, progesterone receptor, or Her2 status. We used an independent set of 15 normal (from non-cancer individuals) and 30 breast cancer samples (grade 3), to analyze the expression of various components of activin-A signaling pathway. As summarized in Table 1 and shown in Figure 1a, we observed upregulation of various components of activin-A signaling pathway including *INHBA*, *SMAD2*, and activin type II receptor. Interestingly, some of the negative regulators of activin signaling pathway such as follistatin, *β-glycan*, *IGSF1*, and *IGSF10* showed downregulation in breast tumors compared with normal samples. Furthermore, although *TGF*-β*1* expression was upregulated, *TGF*β*RII* showed a very significant downregulation in tumors, compared with normal tissues (Supplementary Figure S2). We also analyzed various available breast cancer gene expression data sets (oncomine.org). In accordance with our study, analysis of these data sets shows that activin-A signaling components are frequently deregulated in breast cancers (Supplementary Table S2). TGF-β/activin signaling has been shown to be opposed by BMP signaling pathways in development and disease.^1,25,26^ In congruent with this, BMP isoforms 2, 3, and 6, and various other genes involved in BMP signaling such as *SMAD1*, *ZNF521*, *RGMA*, and *Gremlin1* were found to be downregulated. To confirm our results, we performed immunohistochemistry with another set of 13 normal and 29 tumor samples. As shown in Figure 1b (i), most of the tumors have higher levels of activin-A compared with normals tissues. Activation of activin signalling results in phosphorylation of SMAD2 and SMAD3. In good correlation, breast tumors showed increased phosphoSMAD2 and phosphoSMAD3 levels compared with normal tissues (Figure 1b, ii and iii). In addition, in the same set of tumors, BMP2 staining showed a reduced expression pattern (Figure 1b, iv), compared with normal tissues. We also analyzed the expression of inhibin in some normal and breast tumor samples (data not shown) and found that most of the tumor samples have very low levels of inhibin compared with normal tissues. This suggests that overexpression of *INHBA* results in reduced inhibin expression, possibly due to *INH*β*A* homo-dimerization. In conclusion, our data shows that breast tumors have higher levels of activin-A and low levels of various BMPs, suggesting activation of activin signaling pathway in these tumors.

Further, we also analyzed various publicly available breast cancer clinical data sets (co.bmc.lu.se/gobo/gsa.pl). As represented in Figure 1c (ii), the analysis shows that *INHBA* expression is negatively correlated with the overall survival of grade 3 breast cancer patients. However, there was no significant correlation between *INHBA* expression and overall patient survival, when all the grades are taken together (Figure 1c, i). Further, the analysis revealed that *INHBA* expression correlates negatively with the distant metastasis-free survival of breast cancer patients (Figure 1c, iii and iv). Interestingly, expression of two important negative regulators of activin signaling pathway, *FST* and *TGFBR3*, is progressively reduced from grade 1 to grade 3 (Figure 1d). This suggests that activin signaling may progressively increase from grade 1 to grade 3, even without any significant change in the expression of *INHBA*. On the other hand, expression of *FST* and *TGFBR3* is positively correlated with the distant metastasis-free survival of breast cancer patients (Supplementary Figure S3). All these results suggest that activin-A signaling pathway has an important role in the metastasis and, hence, survival of breast cancer patients.

### Activin-A promotes anchorage-independent growth of breast cancer cells

**Figure 2:**
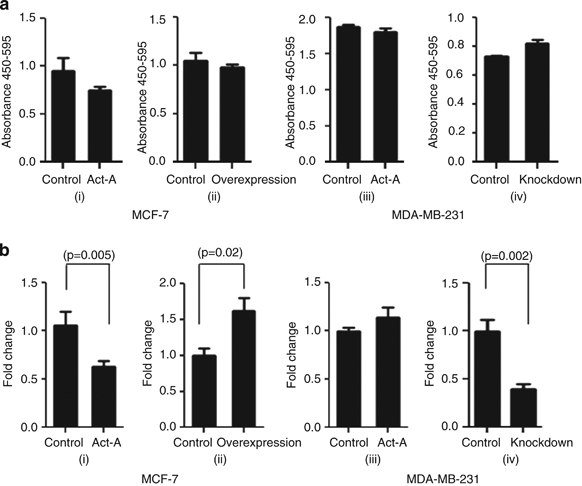
Activin-A promotes anchorage-independent growth but not proliferation of breast cancer cells. (**a**) Bromodeoxyuridine (BrdU) incorporation assay following activin-A treatment of MCF7 (i) and MDA-MB-231 (iii) cells. Overexpression of INHBA in MCF7 cells (ii) and knockdown of INHBA by small hairpin RNA (shRNA) in MDA-MB-231 cells (iv). MCF-7 cells but not MDA-MB-231 cells show mild inhibition in proliferation on activin-A treatment. (**b**) Treatment of MCF-7 cells with activin-A results in reduced colony growth (i), but MCF-7 clones overexpressing activin-A show higher colony-forming ability (ii). Treatment of MDA-MB-231 cells with activin-A in soft agar does not show any effect (iii) and stable knockdown of activin-A in MDA-MB-231 cells results in a significant decrease in colony formation (iv).

In order to understand the role of activin-A in breast tumors, we used MCF-7 and MDA-MB-231 cells as models to study the response of these cell lines to activin-A. Both these cell lines are responsive to activin-A treatment (addition of recombinant human activin-A) as determined by an increase in phosphoSMAD2 levels (Supplementary Figure S6). Although these two cell lines represent different categories of breast tumors, one being estrogen receptor positive and the other being triple negative, there was no difference in the expression of activin in these tumors, as mentioned in the previous section. Most importantly, we chose these cells based on their *INHBA* expression levels for overexpression and knockdown studies. We evaluated the effect of activin-A signaling on proliferation and anchorage-independent growth of these cell lines in monolayer and anchorage-independent culture conditions. On activin-A treatment MCF-7 cells showed a decrease, whereas MDA-MB-231 cells showed no change in proliferation as assessed by bromodeoxyuridine incorporation assay (Figure 2a, i and iii). We cloned and overexpressed *INHBA* (activin-A) in MCF-7 and knocked down its expression using small hairpin RNA in MDA-MB-231 cells (Supplementary Figure S7). Stable overexpression or knockdown of activin-A in MCF-7 and MDA-MB-231 cells, respectively, did not affect their proliferation (Figure 2a, ii and iv). In contrast to activin-A treatment, which resulted in a decrease in proliferation of MCF-7 cells, activin A-expressing clones may represent cell populations that have overcome the growth inhibitory action of activin-A. In addition, given the heterogeneous nature of cell lines, there may be a subpopulation that is refractory to growth inhibition by activin. It is likely to be that during the process of selection, cells that are sensitive to growth inhibition by activin get eliminated and cells refractory to the growth inhibitory signals of activin-A get selected, which mimics the actual tumor progression. Interestingly, even treatment of activin-A-overexpressing MCF-7 cells with TGF-β did not result in any inhibition in their proliferation (Supplementary Figure S8). On activin-A treatment, MCF-7 cells showed a decrease, whereas there was no significant change in number of colonies formed by MDA-MB-231 cells, in soft agar (Figure 2b, i and iii). MDA-MB-231 cells are considered to be aggressive with enriched CD44^high^ and CD24^low^ population. Hence, addition of Activin-A may not result in any further increase in number of colonies. Intrestingly, MCF-7 cells overexpressing activin-A showed an increase, whereas its knockdown showed a decrease in the number of colonies formed by MCF-7 and MDA-MB-231 cells, respectively (Figure 2b, ii and iv). In conclusion, activin-A promotes anchorage-independent growth of cancer cells in a context-dependent manner.

### Activin-A induces epithelial–mesenchymal transition in breast cancer cells

**Figure 3:**
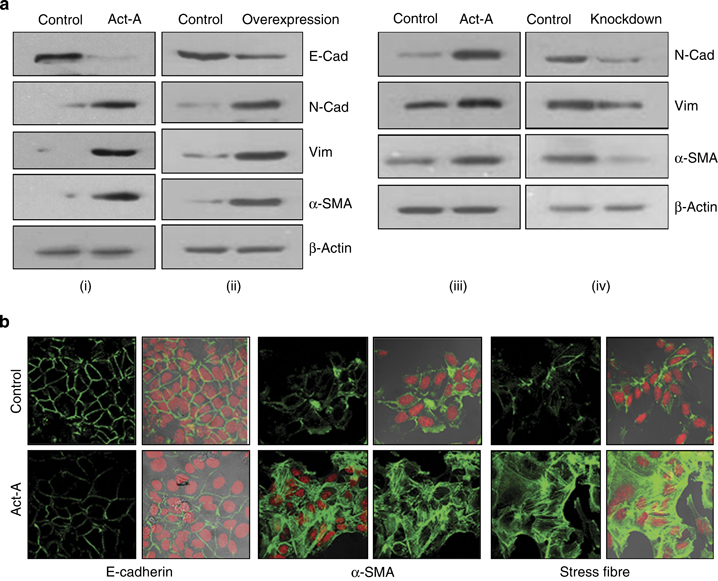
Activin regulates epithelial–mesenchymal transition (EMT) markers in breast cancer cells. (**a**) Western blot analyses showing activin-A treatment (i) or its stable overexpression (ii) in MCF7 cells, or activin A treatment (iii) or knockdown (iv) in MDA-MB-231 cells regulates EMT markers (it is noteworthy that MDA-MB-231 cells do not express E-cadherin). (**b**) Confocal microscope images of activin-A-treated MCF7 cells show cytoskeletal changes marked by decreased E-cadherin, increased α-smooth muscle actin (SMA) and stress fibre formation (phalloidin-fluorescein isothiocyanate staining).

Epithelial–mesenchymal transition (EMT), a process in which epithelial cells lose some of their characteristics and acquire properties of mesenchymal cells, has been proposed to have a pivotal role in invasion/metastasis of cancer cells. Unlike TGF-β, the role of activin-A in this regard is unknown. Western blot analysis shows that treatment or overexpression of activin-A leads to a decrease in the expression of E-cadherin and an increase in the expression of various mesenchymal markers in MCF-7 cells (Figure 3a, i and ii). Interestingly, we could also observe a mesenchymal phenotype in activin-A-overexpressing MCF-7 cells (Supplementary Figure S9). EMT is also marked by stress fiber formation associated with changes in the cytoskeleton. Confocal microscopy indicated that treatment with activin-A leads to downregulation of E-cadherin, induction of α-smooth muscle actin, and stress fibre formation in MCF-7 cells (Figure 3b). Similar results were observed on treatment or knockdown of activin-A in MDA-MB-231 cells (Figure 3a, iii and iv). In conclusion, similar to TGF-β, activin-A also is an inducer of EMT in cancer cells.

### Activin-A promotes migration and invasion of MDA-MB-231 breast cancer cells

**Figure 4:**
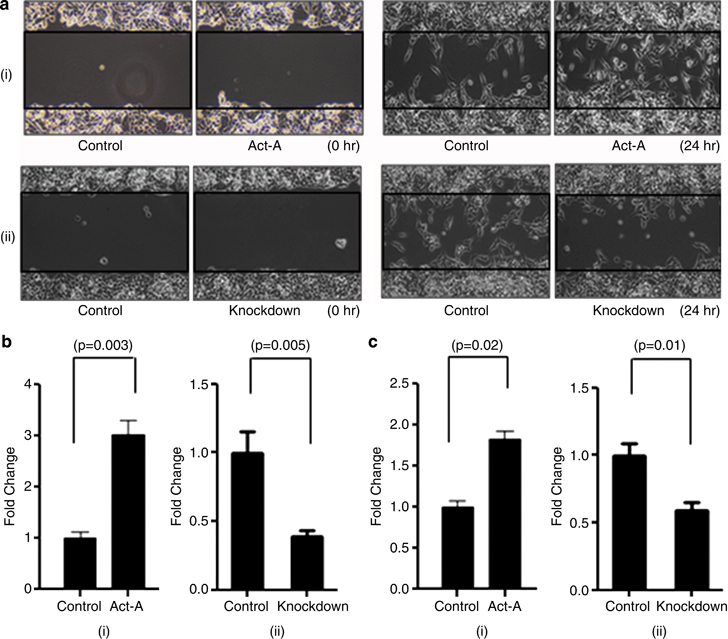

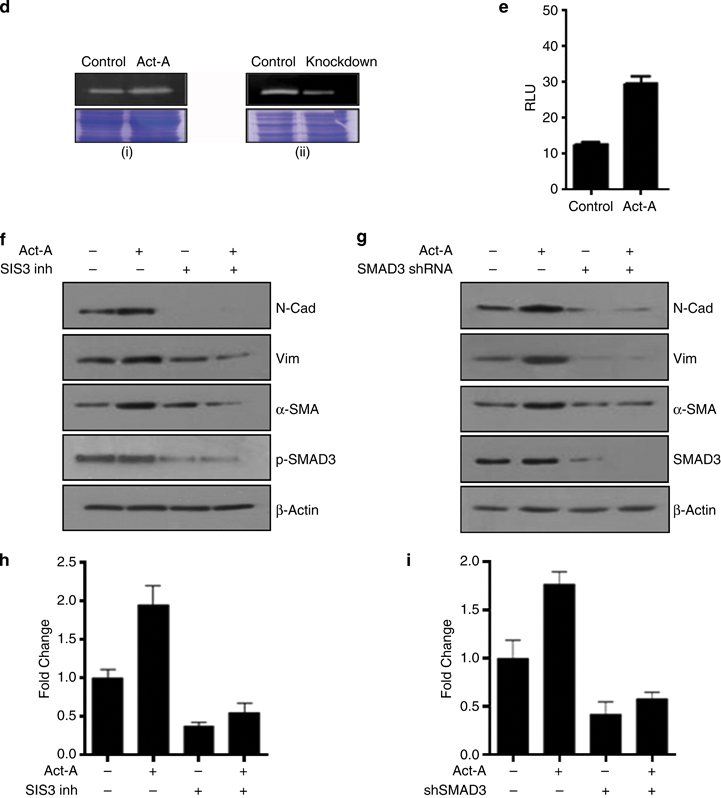
Activin promotes migration and invasion of breast cancer cells. (**a** and **b**) Activin-A treatment (i) increases, whereas its stable knockdown (ii) decreases migration of MDA-MB-231 cells as shown by the scratch assay and transwell migration assay, respectively. (**c**) Matrigel invasion assay shows that activin-A treatment (i) increases, whereas its knockdown (ii) decreases invasion of MDA-MB-231 cells. (**d**) Zymography using MDA-MB-231 cell supernatant shows that activin-A treatment increases (i) and its knockdown decreases (ii) active matrix metalloproteinase-2 (MMP2) levels. (**e**) Luciferase reporter assay in HEK 293T cells shows that activin-A regulates MMP2 promoter activity. (**f** and **g**) Activin-A regulation of epithelial–mesenchymal transition (EMT) markers is inhibited using SMAD3 inhibitor or by stable knockdown of SMAD3 using small hairpin RNA (shRNA) in MDA-MB-231 cells. (**h** and **i**) Activin-A-induced increase in invasion in MDA-MB-231 cells is abrogated in the presence of SMAD3 inhibitor or stable knockdown of SMAD3.

EMT has been associated with migratory and invasive behavior of cancer cells.^27^ Hence, we studied whether activin-A has any effect on migratory and invasive behavior of MDA-MB-231 cells. Wound-healing (scratch) assay showed that activin-A treatment of MDA-MB-231 cells promotes their migration, whereas knockdown of activin-A results in decreased migration of these cells (Figure 4a, i and ii). We also performed transwell migration assay and observed that treatment of activin-A increases, whereas stable knockdown of activin-A decreases migration of MDA-MB-231 cells (Figure 4b, i and ii). To assess whether activin-A affects the invasive behavior of MDA-MB-231 cells, we performed Matrigel invasion assay and observed that treatment with activin-A increases, whereas knockdown of activin-A reduces invasive potential of MDA-MB-231 cells (Figure 4c, i and ii). Next, we performed zymography to analyze MMP2 and MMP9 activity. The results show that activin-A treatment increases, whereas its knockdown decreases MMP2 activity in MDA-MB-231 cells (Figure 4d, i and ii). Further, we used Luciferase-conjugated MMP2 promoter and assayed its inducibility by activin-A. Activin-A treatment of HEK-293T cells transfected with the reporter construct showed a significant increase in the promoter activity (Figure 4e). These results suggest that activin-A can activate transcription of MMP2 and promote migration and invasion of breast cancer cells.

Many recent studies have shown that SMAD3 has an important role in TGF-β-induced EMT, migration, and invasion.^28,29^ Hence, we used a specific inhibitor (SIS3) and small hairpin RNA-mediated stable knockdown of SMAD3 in MDA-MB-231 cells. The results show that ablation of SMAD3 activity leads to blockade of activin-A-induced EMT and invasion in these cells (Figure 4f–i). Together, these results suggest that activin-A-induced phenotype is dependent on SMAD3 signaling in MDA-MB-231 cells.

### Activin-A promotes tumorigenicity of breast cancer cells

**Figure 5:**
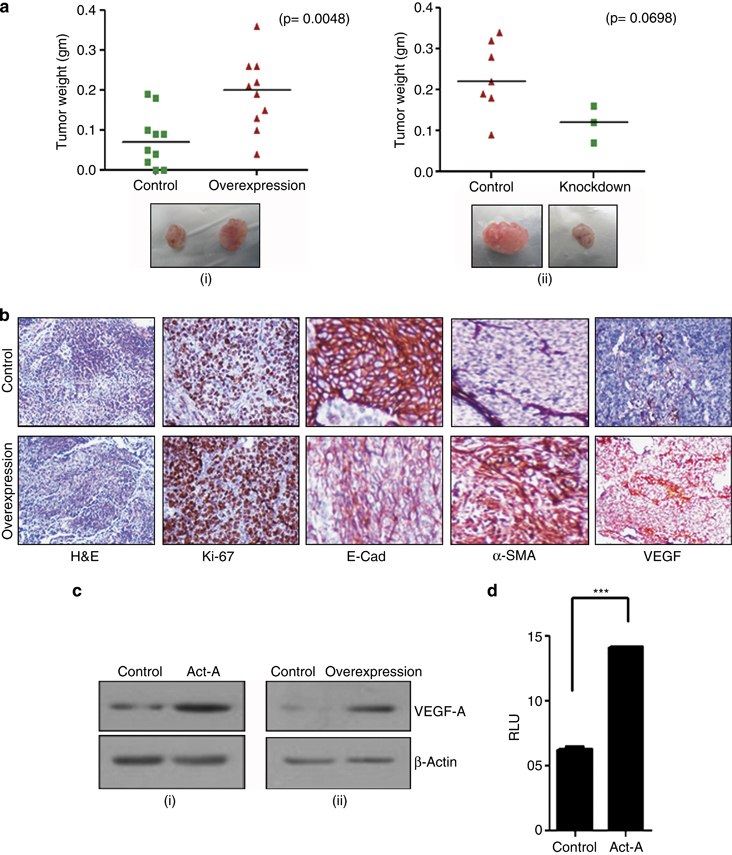

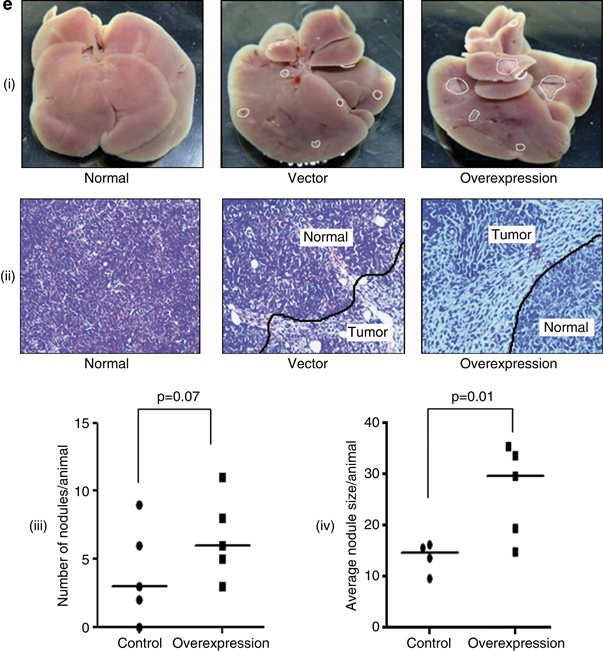

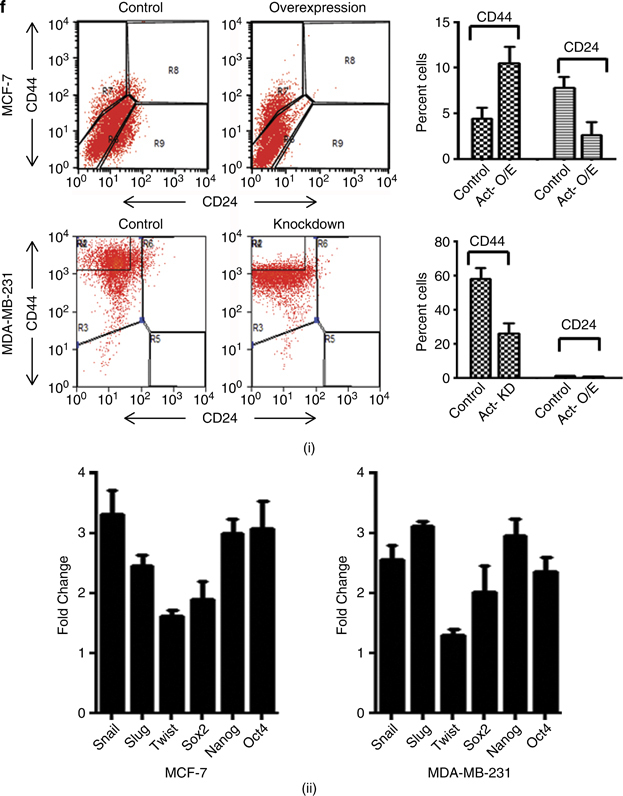
Activin promotes tumorigenicity of breast cancer cells in immunocompromised mice. (**a**) Stable overexpression of activin-A in MCF-7 enhances (i), whereas stable knockdown of activin-A in MDA-MB-231 cells (ii) reduces their tumor-forming ability in nude mice. Shown below are the representative images of the tumors formed s.c. (**b**) Immunohistochemical analysis of MCF-7-overexpressing tumors shows EMT-like changes and higher ki-67 index. (**c**) Treatment of MCF-7 cells with activin-A (i) or overexpression of activin-A in MCF-7 cells (ii) results in increased levels of vascular endothelial growth factor-A (VEGF-A). (**d**) Luciferase reporter assay in HEK 293T cells shows that activin-A regulates VEGF promoter activity. (**e**) Tail vein injection of activin-A-overexpressing MCF-7 cells shows better tumor-forming ability in the livers of nude mice (i). Normal is shown here as the reference from an animal without injection of any cells. The panel below (ii) shows the hematoxylin and eosin (H&E) staining of the liver tissue sections. The graph (iii) and (iv) shows number and size of nodules formed in the liver per animal. (**f**) CD44^high/^CD24^low^ fluorescence-activated cell sorting (FACS) analysis of activin-A-overexpressing or knockdown cells shows that activin-A influences stemness of breast cancer cells (i). Quantitative PCR analysis shows that treatment of MCF-7 or MDA-MB-231 cells with recombinant activin-A induces various markers of stemness (ii).

The process of tumor formation is highly complex. TGF-β signaling has been shown to have an important role in the establishment of tumors *in vivo.* ^30–32^ Hence, we wanted to investigate how activin-A would affect the tumor-forming ability of cancer cells *in vivo*. We injected activin-A-overexpressing MCF7 cells in the flank of immunocompromised mice and followed till the tumors reached to a prominent size. Our results show that activin-A-overexpressing MCF-7 cells have better tumor-forming ability in comparison with control cells (Figure 5a, i). We also injected only half a million activin-A knockdown MDA-MB-231 and control cells (optimal cell number generally used is two million) subcutaneously in nude mice. Although 7 out of 10 animals formed tumors in control mice, only 3 out of 10 animals injected with activin-A knockdown cells could lead to tumor formation (Figure 5a, ii). Significant differences in the weight of the tumors were also observed. We also performed immunohistochemistry on the tumors formed by MCF7 cells (Figure 5b). We observed that MCF7 overexpressing activin-A tumors have higher Ki-67 percentage (~80%) as compared with control tumors (~50%). In addition, staining for various EMT markers confirmed the mesenchymal state of tumors formed by activin-A-overexpressing MCF-7 cells. Recruitment of blood vessels or *de-novo* formation of blood vessels can influence the tumor growth *in vivo*. Hence, we investigated the expression of VEGF, which is known to promote angiogenesis. Activin-A treatment or its stable overexpression in MCF-7 cells induced VEGF expression (Figure 5c, i and ii). Further, we performed Luciferase reporter assay to study the regulation of VEGF promoter by activin-A. Activin-A treatment of HEK-293T transfected with Luciferase-conjugated VEGF promoter showed that activin-A induces activity of VEGF promoter significantly (Figure 5d). We also performed tail vein injections with activin-A-overexpressing MCF-7 cells. Even though we did not find a significant difference in the number of nodules formed, we observed that activin-A-overexpressing cells formed much bigger nodules as compared with the control cells (Figure 5e, iii and iv). This suggests that growth of colonized MCF-7 cells is promoted by activin-A expression. Tumor-forming ability of cancer cells and aggressiveness of various cancers has been associated with the presence of cells having stem-like phenotype.^33,34^ Activin-A has been shown to regulate expression of various stemness markers such as Nanog, Sox2, and Oct4 in various cells.^35,36^ Hence, to investigate whether activin-A expression affects stemness of breast cancer cells, we analyzed population of CD44^high^ and CD24^low^ cells. Fluorescence-activated cell sorting analysis of activin-A-overexpressing MCF-7 cells and activin-A knockdown MDA-MB-231 cells shows that activin-A expression leads to enrichment of breast cancer stem-like cells (Figure 5f, i). In addition, treatment of MCF-7 and MDA-MB-231 cells with activin-A leads to an increase in expression of several stemness markers (Figure 5f, ii). Taken together, these results suggest that activin-A has multiple effects on tumor establishment and progression *in vivo*.

## Discussion

Activin-A, a member of the TGF-β superfamily, binds to its cognate receptor and activates SMAD2/3 signaling pathway. However, unlike TGF-β, the role of activin in cancer is not well known. Here we show that high-grade breast tumors have activation of activin-A signaling pathway. Interestingly, we did not observe much significant change in the expression of TGF-β ligands. We also demonstrate that many components and regulators of activin-signaling pathway are deregulated, favoring the activation of this signaling pathway in these tumors. Our results show that breast tumors have higher phosphoSMAD2 and phosphoSMAD3 levels as compared with normals, which shows that this signaling pathway is active in these tumors. Cellular response towards various environmental stimuli is a highly complex process. Response towards a factor is modulated by the presence or the absence of many other molecules, which is further governed by cellular context. Here we show that BMPs and various components, as well as regulators, of this signaling pathway are deregulated, favoring suppression of BMP signaling in breast tumors. In addition, breast tumors revealed significant downregulation of SMAD1, which acts as a downstream mediator of the BMP signaling pathway. Downregulation of SMAD1 in breast tumor cells will abrogate any BMP signaling (locally or at a distant site) in these cells. BMP signaling pathway has been shown to have an antagonistic role to TGF-β/activin signaling in various physiological and pathological conditions.^37,38^ In addition, recent studies have shown that BMP signaling has a tumor-suppressive function in cancers.^39^ Hence, hyperactivation of activin-A signaling and loss of BMP signaling may have a critical role in the clinical outcome of breast cancers. We show that *INHBA* expression correlates negatively with the overall survival of high-grade breast tumors and metastasis-free survival of breast cancer patients. In agreement with these results, *FST* and *TGFBR3* expression confers a good prognosis for breast cancers. It suggests that activin-A signaling has an important role in the dissemination of breast cancer cells and, hence, may determine the outcome of the disease. We show that overexpression or knockdown of activin-A affects anchorage-independent growth of breast cancer cells. We also show that activin-A induces EMT and promotes migration and invasion in breast cancer cells. Primarily, the SMAD2/3 signaling pathway inhibits proliferation of normal epithelial cells and, hence, is considered to have a tumor-suppressor function. Similar to TGF-β, proliferation of normal or tumor cells in the earlier stages of tumor development may be inhibited by activin-A. However, it is interesting to note that this signaling pathway still remains intact in majority (~98%) of the breast cancers.^40^ With the disease progression, these cells may become refractory to the growth inhibitory effect of activin-A signaling, as exemplified in prostate cancer. Activin has been shown to inhibit proliferation of LNCaP and DU145 (low- and moderate-grade PCa) but not PC3 cell line (high grade). Interestingly, circulating levels of activin-A were demonstrated to increase significantly in metastatic prostate and breast cancers.^41,42^ In addition, SMAD deletions are observed only in 1–2% of breast cancers.^40^ In other words, this signaling pathway remains intact in most of the breast cancers. Given the abundant expression of activin-A in tumor cells and the presence of an intact signaling pathway, it is reasonable to believe that these cells have acquired resistance to the growth inhibitory effect of activin-A. Moreover, it is quite comprehensible that SMADs may then have an important role in tumor progression. Although TGF-β is well known to induce EMT in various cell lines, not much is known about activin-A in this regard. EMT is considered to be a prerequisite for tumor cells to migrate and invade into neighboring tissues, and hence lead to metastasis. In recent times, even nodal, another member of the TGF-β superfamily that signals through the activin receptors has been reported to promote cancer progression. Our results demonstrate that activin-A-induced expression of EMT markers and invasion is SMAD3 dependent. SMAD3 has been shown to promote growth of breast cancer cells in nude mice.^43^ However, activin-A may also activate non-SMAD signaling pathways, which may contribute to its pro-tumorigenic actions. We observed that activin affects tumor formation and colonization of tumor cells in nude mice. The data suggest that activin-A expression affects the establishment of tumors, which is a very complex process. Establishment of tumors or metastasis is also influenced by interaction between tumor cells and the microenvironment. Hence, factors that can modulate the tumor microenvironment can have a key role in cancer progression. In this context, activin may also be important in the establishment of metastases from disseminated cells by modifying the tumor niche.^22^ One of the factors known to be important for tumor growth is VEGF, which leads to recruitment of blood vessels, hence providing nourishment to the tumor cells. As we have demonstrated here, activin-A induces VEGF expression and may influence the proliferation of the cancer cells *in vivo*. It may, to some extent, also explain how activin overexpression promotes proliferation of tumor cells *in vivo*. It will be interesting to evaluate how activin-A expression would affect the response of breast cancer patients to various conventional treatments. Activin-A is known to have an important role in maintenance of stem cell phenotype^11^ and stemness.^36^ In addition, EMT has been shown to induce drug resistance and stem cell-like phenotype in cancer cells.^44^ It has also been shown that tumors that are more aggressive are less differentiated and vice versa. We show that activin-A expression affects the stemness of breast cancer cells and hence may contribute to the aggressiveness of the disease. Our study highlights the importance of activin signaling in the progression of breast tumors. Administration of a circulating dominant-negative type II TGF-β receptor in mice has been shown to prevent metastasis of breast tumors.^45^ However, our study emphasizes the role of activins and the expression pattern of these ligands should be considered from a clinical perspective. It is possible that different tumors may use either TGF-β or activin in a context-dependent manner. Hence, it is important to carefully examine the expression of these ligands while designing strategies to block their actions. Although most studies in the past have focussed on the role of TGF-β, this study emphasizes activin-A’s role in the progression of breast tumors. Interestingly, many other cancers have been reported to have increased expression of activins, suggesting that activation of the activin signaling pathway may be widely involved in carcinogenesis. As activins have multiple roles in physiological context, their role in cancer may be equally diverse. Importantly, our study emphasizes the role of SMAD pathway in the progression of breast tumors and targeting this pathway may be a useful strategy in the treatment of breast cancers. In conclusion, we show that activin-A induces EMT, promotes invasion, and enhances the tumor-forming ability and metastatic growth of breast cancers.

## Figures and Tables

**Table 1:** qPCR analysis of normal and breast tumor samples shows that components and regulators of activin-A signaling pathway are deregulated in breast tumors

*Gene*	*Function*	*Median fold change*	P*-value*
*INHBA*	A member of TGFβ superfamily of cytokines, activating SMAD2/3 signaling pathway	11.31	<0.0001
*INHA*	Binds to ACTRII and acts as a negative regulator of activin signaling	No change	—
*ACVR2A*	Cell surface serine–threonine kinase receptor of activin signaling	2.44	0.0007
*SMAD2*	Acts as a mediator of activin signaling pathway	2.02	0.0453
*FST*	Binds directly to activin and prevents its binding to the receptor	−1.81	0.0050
*TGFBR3*	Acts as a co-receptor for inhibin binding to ACTRII	−6.49	<0.0001
*IGSF1*	Binds to activin directly and prevents its binding to the receptor	−2.75	0.0001
*IGSF10*	Binds to activin directly and prevents its binding to the receptor	−25.63	<0.0001
*BMP2*	A member of the TGF-β superfamily of cytokines, activating SMAD1/5 signaling pathway	−5.2	0.0003
*BMP3*	Binds ACTRIIB, however, has no defined type I receptor	−5.81	0.0254
*BMP6*	A member of the TGF-β superfamily of cytokines, activating SMAD1/5 signaling pathway	−3.07	0.0012
*RGMA*	Acts as a co-receptor in BMP signaling	−8.11	<0.0001
*SMAD1*	Acts as a signal transducer of BMP signaling	−2.60	0.0217
*ZNF521*	Acts as a co-transcription factor in association with SMAD1	−2.65	<0.0001
*GDF10*	Also known as BMP3B and binds to ACTRII with no defined type I receptor	−13.36	<0.0001
*GREMLIN1*	Binds to BMPs directly and prevents their binding to the receptor	2.25	0.0060
*TWSG1*	Forms a ternary complex with BMPs along with chordin	2.12	0.0019

Abbreviations: BMP, bone morphogenetic protein; qPCR, quantitative PCR; TGF-β, transforming growth factor-β.
