# Supplementary figures and images for: Activin-A signaling promotes epithelial–mesenchymal transition, invasion, and metastatic growth of breast cancer
Source: NPJ Breast Cancer. 2015 Aug 12;1:15007–. doi: 10.1038/npjbcancer.2015.7 (PMC5515205; doi:10.1038/npjbcancer.2015.7)

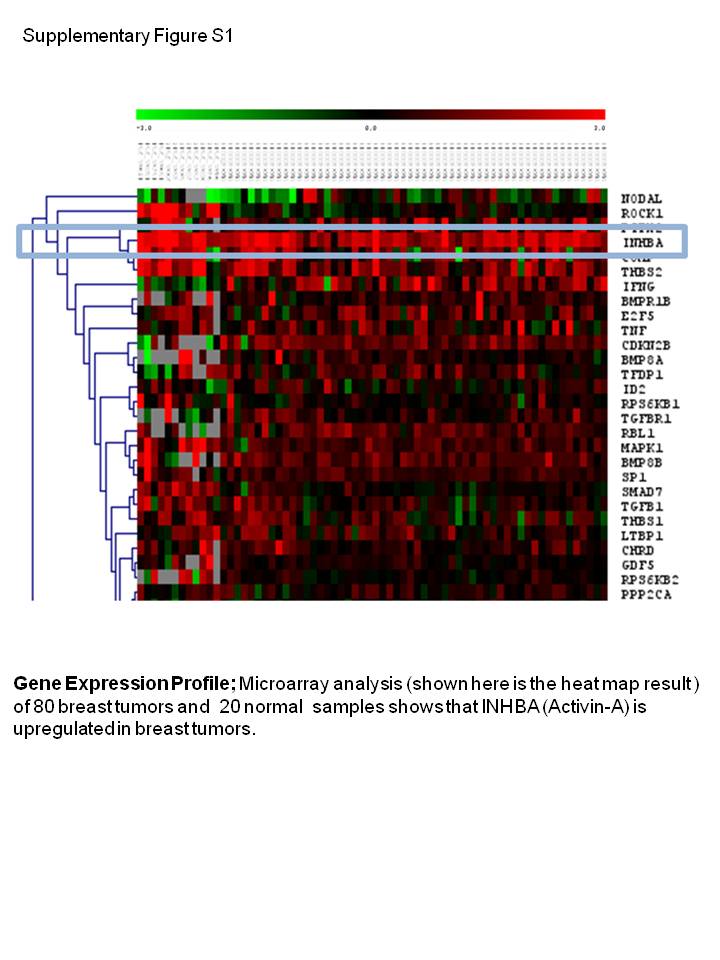

Supplement: Supplementary Figure 1 [file npjbcancer20157-s1.jpg]

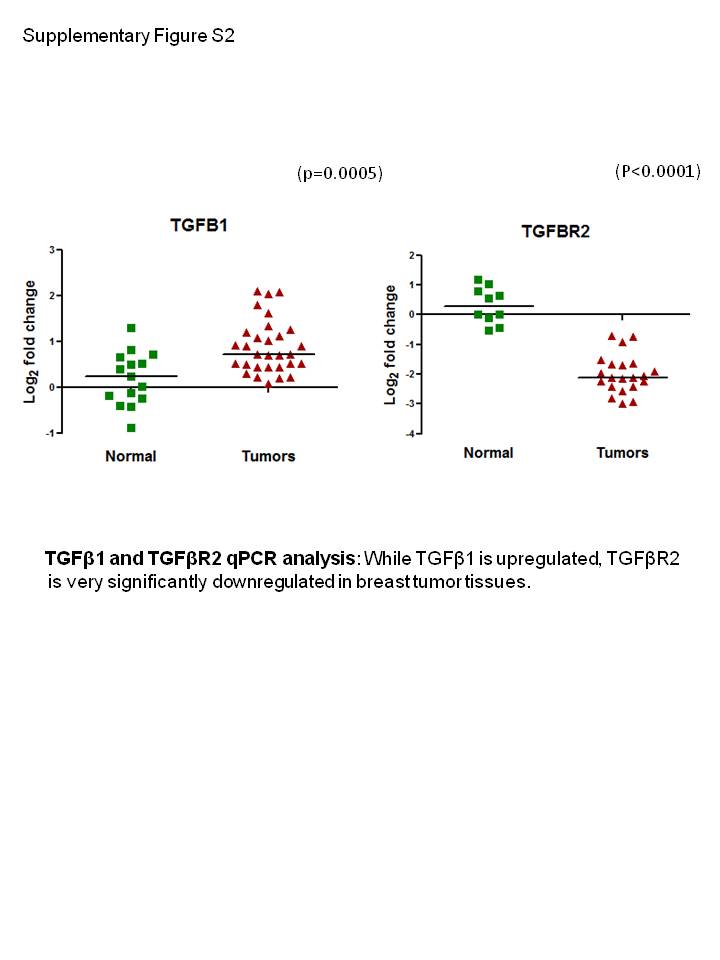

Supplement: Supplementary Figure 2 [file npjbcancer20157-s2.jpg]

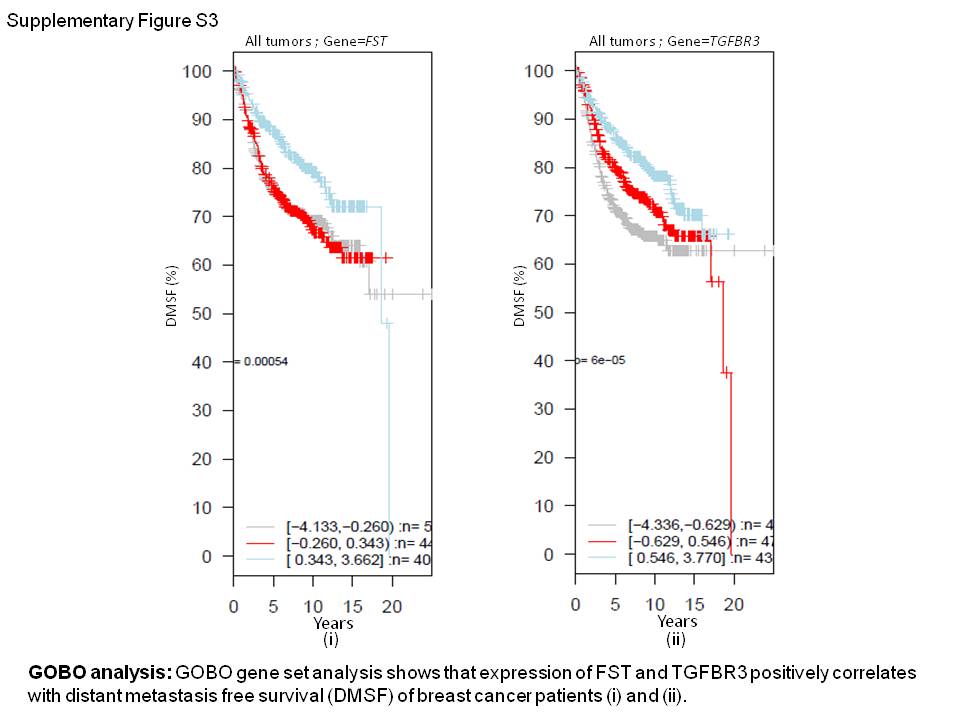

Supplement: Supplementary Figure 3 [file npjbcancer20157-s3.jpg]

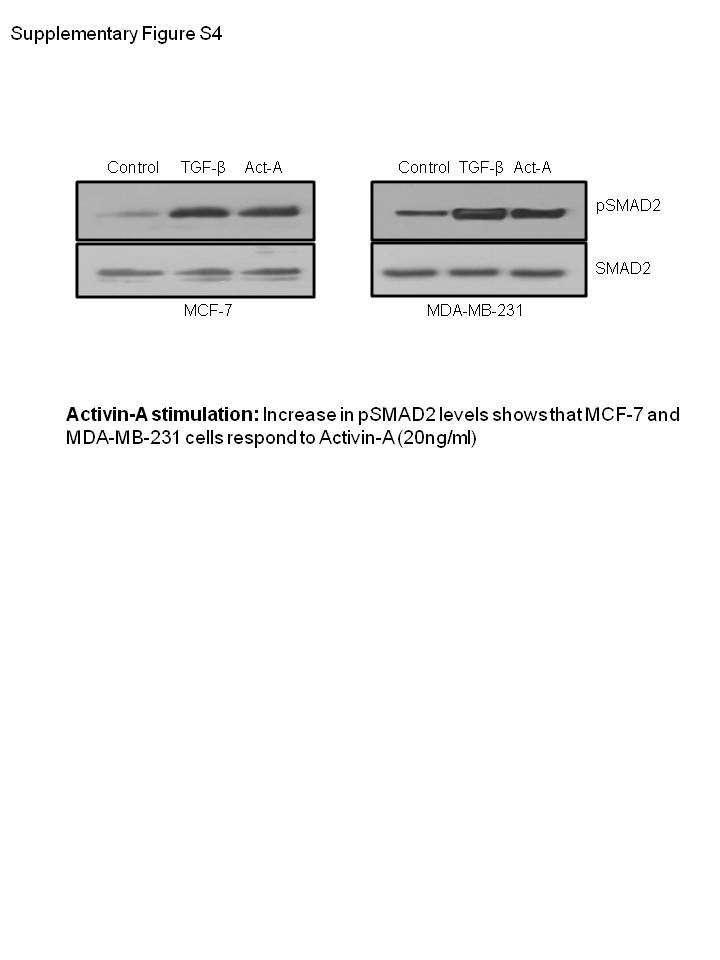

Supplement: Supplementary Figure 4 [file npjbcancer20157-s4.jpg]

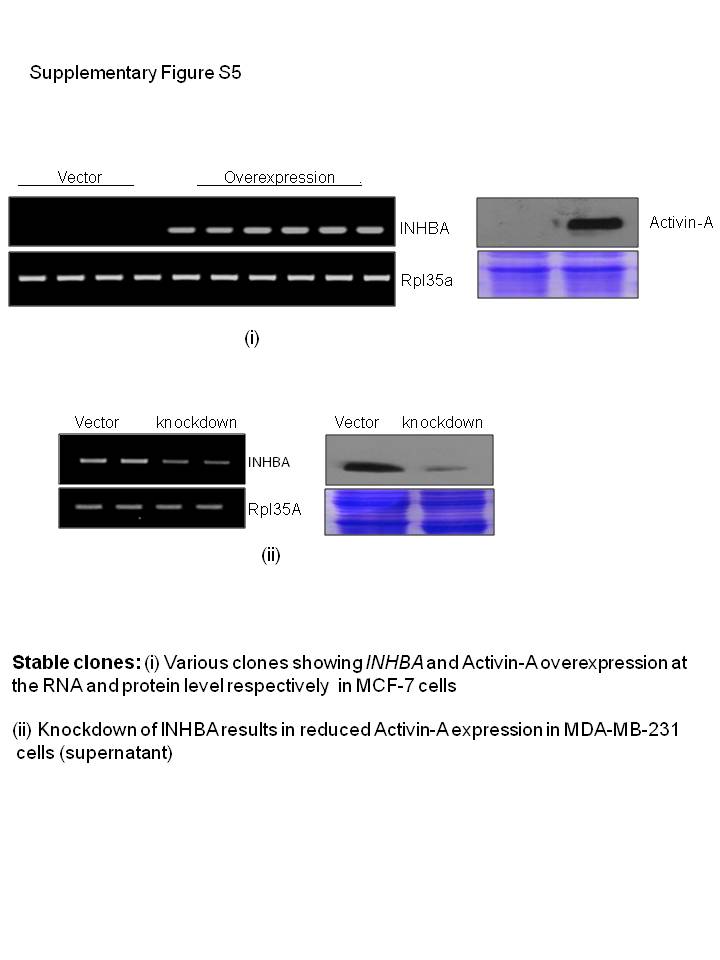

Supplement: Supplementary Figure 5 [file npjbcancer20157-s5.jpg]

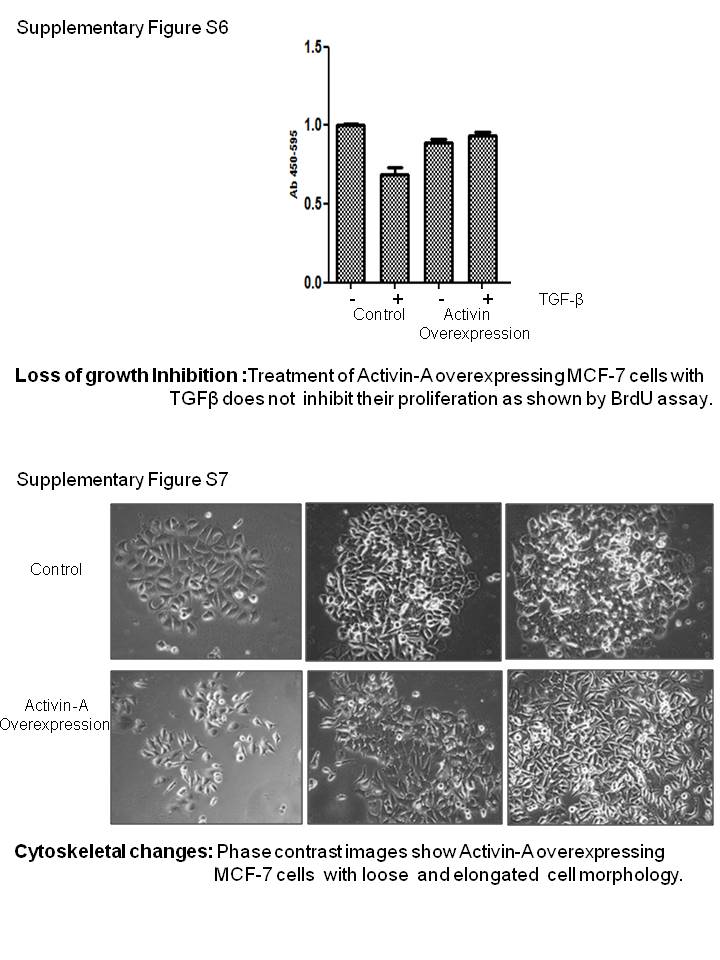

Supplement: Supplementary Figure 6 and 7 [file npjbcancer20157-s6.jpg]
