# Supplementary material for: Activin-A signaling promotes epithelial–mesenchymal transition, invasion, and metastatic growth of breast cancer
Source: NPJ Breast Cancer. 2015 Aug 12;1:15007–. doi: 10.1038/npjbcancer.2015.7 (PMC5515205; doi:10.1038/npjbcancer.2015.7)
Supplement: Supplementary Tables [file npjbcancer20157-s7.doc]

**Supplementary Table S1:** Primer sequence of indicated genes used in qPCR analysis

| **Gene Name** | **Forward Primer** | **Reverse Primer** |
| --- | --- | --- |
| *INHBA* | AATCTCGAAGTGCAGCGTCT | GGAGAACGGGTATGTGGAGA |
| *INHA* | TGCGTGTATGCTGGGATG | GGAAGAGGAGGATGTCTCCC |
| *ACVR2A* | TGTCACCATAACACGGTTCAA | GCGTTTGCCGTCTTTCTTAT |
| *SMAD2* | ACAGCCAGTTACTTACTCAG | GATTGCACTATCACTTAGGC |
| *FST* | ACTCCTCCTTGCTCAGTTCG | ACTCCTCCTTGCTCAGTTCG |
| *TGFBR3* | AGTTCACACAGTGCACCAGG | TGAGCAGGCTGAAGTGACTG |
| *IGSF1* | TCTGTCCAGGGTCATGGG | CTGGAGGAGCTCACTGGAGA |
| *IGSF10* | CCCATTTTGTTTCTTGCCAC | TTACCATCCAAGGCTGTGGT |
| *BMP2* | TTTTCCCACTCGTTTCTGGT | GCTAGACCTGTATCGCAGGC |
| *BMP3* | TCAGGCTGATGTTTCCTAGC | ACACGGTTCGCAGCTTTC |
| *BMP6* | CAGTCCTTGTAGATGCGGAA | CATGAGCTTTGTGAACCTGG |
| *RGMA* | TGCACTTGAGGATCTTGCAC | CTAGTGGTAACAGGCCGAGC |
| *SMAD1* | CCAAATGCAAAAGGACAGCA | CCTTTACTCTGCTCCCTGTCTT |
| *ZNF521* | AGAGTCAAAGGTCTGGCTGC | CCCAGTCGGATGAGAAGAAG |
| *GDF10* | TTGCATGGAAGTCAGGTTGA | GTCCACATGCACAGGCTCTA |
| *GREMLIN1* | GTGTTTGGACAAATTCGCCT | TTTTGATCCAGTGCTCTCCC |
| *TWSG1* | GGAAGAGAGAAGGGATCGGT | GAGTGCATGCTGTGTCTTGG |
| *HPRT1* | ACCCTTTCCAAATCCTCAGC | TCCTCCTCCTGAGCAGTCA |

**Supplementary Table S2:** Oncomine data Analysis shows over-expression of Activin-A and its signaling pathway components in breast tumors (FC=Fold change)

| **Gene** | **Analysis** | **Source** | **FC** | **p-value** |
| --- | --- | --- | --- | --- |
| *INHBA* | Invasive Ductal Breast Carcinoma Stroma: Normal | Karnoub Breast | 10.44 | 1.17E-4 |
|  | Ductal Breast Carcinoma: Normal | Sorlie Breast | 6.45 | 4.81E-7 |
|  | Lobular Breast Carcinoma: Normal | Perou Breast | 5.82 | 9.95E-4 |
|  | Invasive Mixed Breast Carcinoma: Normal | Radvanyi Breast | 3.9 | 3.53E-4 |
|  | Invasive Lobular Breast Carcinoma: Normal | Radvanyi Breast | 2.76 | 9.90E-4 |
|  | Invasive Ductal Breast Carcinoma: Normal | Radvanyi Breast | 2.70 | 8.54E-4 |
|  | Ductal Breast carcinoma in-situ Stroma: Normal | Ma Breast 4 | 21.04 | 8.21E-11 |
|  | Invasive Ductal Breast Carcinoma Stroma: Normal | Ma Breast 4 | 21.12 | 8.33E-6 |
|  | Ductal Breast Carcinoma: Normal | Richardson Breast 2 | 8.94 | 2.04E-12 |
|  | Invasive Ductal breast carcinoma: normal | Turashvili Breast | 9.75 | 2.00E-5 |
|  | Invasive Breast Carcinoma: Normal | TCGA Breast | 8.35 | 1.22E-44 |
|  | Invasive Ductal Breast carcinoma: Normal | TCGA Breast | 7.89 | 5.83E-48 |
|  | Invasive Lobular Breast Carcinoma: Normal | TCGA Breast | 8.38 | 2.31E-24 |
| *ACVR1B* | Invasive Ductal Breast Carcinoma: Normal | Turashvili Breast | 1.73 | 0.01 |
|  | Mixed Lobular and Ductal Breast Carcinoma: Normal | TCGA Breast | 1.31 | 7.95E-6 |
|  | Ductal Breast Carcinoma: Normal | Richardson Breast2 | 1.4 | 0.002 |
|  | Intra-ductal cribiform Breast Adenocarcinoma: Normal | TCGA Breast | 1.72 | 0.003 |
|  | Ductal Breast Carcinoma: Normal (Copy Number Gain) | TCGA Breast 2 |  |  |
|  | Invasive Breast Carcinoma Stroma: Normal | Finak Breast | 1.44 | 2.04E-6 |
| *ACVR2B* | Invasive Ductal and Lobular Carcinoma: Normal | TCGA Breast | 1.51 | 0.002 |
|  | Intraductal Cribiform Breast Adenocarcinoma: Normal | TCGA Breast | 2.34 | 0.03 |
|  | Invasive Ductal Breast carcinoma Stroma: Normal | Karnoub Breast | 1.48 | 0.01 |
| *SMAD2* | Invasive Mixed Breast Carcinoma: Normal | Radvanyi Breast | 2.50 | 0.004 |
|  | Invasive Lobular Breast Carcinoma: Normal | Radvanyi Breast | 2.29 | 0.004 |
|  | Invasive Breast Carcinoma Stroma: Normal | Finak Breast | 3.98 | 4.22E-16 |
|  | Invasive Lobular Breast Carcinoma: Normal | Turashvili Breast | 2.02 | 0.03 |
|  | Intra-ductal Cribiform Breast Adenocarcinoma: Normal | TCGA Breast | 1.53 | 6.54E-4 |
| *SMAD3* | Lobular Breast Carcinoma: Normal | Perou Breast | 2.02 | 0.007 |
|  | Invasive Ductal Breast Carcinoma: Normal | Zhao Breast | 1.39 | 4.68E-7 |
|  | Lobular Breast Carcinoma: Normal | Zhao Breast | 1.53 | 1.51E-7 |
|  | Invasive Ductal Breast carcinoma Epithelia: Normal | Ma Breast 4 | 1.87 | 0.01 |
