# Supplementary material for: Activin-A signaling promotes epithelial–mesenchymal transition, invasion, and metastatic growth of breast cancer
Source: NPJ Breast Cancer. 2015 Aug 12;1:15007–. doi: 10.1038/npjbcancer.2015.7 (PMC5515205; doi:10.1038/npjbcancer.2015.7)
Supplement: Supplementary Information [file npjbcancer20157-s8.doc]

**Supplementary figures:**

**Figure S 1: Gene Expression Profile;** Microarray analysis (shown here is the heat map result) of 80 breast tumors and 20 normal samples shows that *INHBA* (Activin-A) is upregulated in breast tumors.

**Figure S 2: TGFβ1 and TGFβR2 qPCR analysis;** while *TGF*β*1* is upregulated, *TGF*β*R2* is significantly downregulated in breast tumor tissues.

**Figure S 3: *FST* and *TGF*β*R3* correlation;** GOBO gene set analysis shows that *FST and TGF*β*R3* expression inversely correlates with the distant metastasis free survival (DMSF) of breast cancer patients (i and ii).

**Figure S 4: Activin-A stimulation:** Increase in pSMAD2 levels shows that MCF-7 and MDA-MB-231 cells respond to Activin-A (20ng/ml).

**Figure S 5: Stable clones:** (i) Various clones showing *INHBA* and Activin-A overexpression at the RNA and protein level respectively in MCF-7 cells

(ii) Knockdown of INHBA results in reduced Activin-A expression in MDA-MB-231 cells (supernatant).

**Figure S 6: Loss of growth inhibition;** BrdU assay shows that treatment of Activin-A overexpressing MCF-7 cells with TGF-β does not result in inhibition of proliferation of these cells.

**Figure S 7: Cytoskeletal changes;** Phase contrast images show Activin-A overexpressing MCF-7 cells with loose and elongated cell morphology.
